# The Emergence of Discrete Perceptual-Motor Units in a Production Model That Assumes Holistic Phonological Representations

**DOI:** 10.3389/fpsyg.2019.02121

**Published:** 2019-09-18

**Authors:** Maya Davis, Melissa A. Redford

**Affiliations:** Department of Linguistics, University of Oregon, Eugene, OR, United States

**Keywords:** speech production, speech acquisition, perceptual-motor integration, mathematical model, whole-word representations, dual lexicon model

## Abstract

Intelligible speakers achieve specific vocal tract constrictions in rapid sequence. These constrictions are associated in theory with speech motor goals. Adult-focused models of speech production assume that discrete phonological representations, sequenced into word-length plans for output, define these goals. This assumption introduces a serial order problem for speech. It is also at odds with children's speech. In particular, child phonology and timing control suggest holistic speech plans, and so the hypothesis of whole word production. This hypothesis solves the serial order problem by avoiding it. When the same solution is applied to adult speech the problem becomes how to explain the development of highly intelligible speech. This is the problem addressed here. A modeling approach is used to demonstrate how perceptual-motor units of production emerge over developmental time with the perceptual-motor integration of holistic speech plans that are also phonological representations; the specific argument is that perceptual-motor units are a product of trajectories (nearly) crossing in motor space. The model, which focuses on the integration process, defines the perceptual-motor map as a set of linked pairs of experienced perceptual and motor trajectories. The trajectories are time-based excursions through speaker-defined perceptual and motor spaces. By hypothesis, junctures appear where motor trajectories near or overlap one another in motor space when the shared (or extremely similar) articulatory configurations in these regions are exploited to combine perceptually-linked motor paths along different trajectories. Junctures form in clusters in motor space. These clusters, along with their corresponding (linked) perceptual points, represent perceptual-motor units of production, albeit at the level of speech motor control only. The units serve as pivots in motor space during speaking; they are points of transition from one motor trajectory to another along perceptually-linked paths that are selected to produce best approximations of whole word targets.

## 1. Introduction

Speech can be experienced as a sequence of discrete sounds, at least among literate adults who have used a phonemic writing system from a young age. Linguistic theory in the west has leveraged this experience. Discrete sound units, such as phonemes, have been used by linguists to great analytic and practical advantage in work on the sound patterns of language. This is because “phonemic theory provides a basis for representing the physiological time functions of speech by discrete symbolic sequences (Peterson and Harary, 1961, p. 140).” Peterson and Harary go on to explain, in the proceedings from the 12th Symposium in Applied Mathematics, that “(an) essential part of this theory is the organization of the phone, a basic phonetic unit, into higher order sets of allophones and phonemes.” They then argue that the basis for treating sounds as discrete symbols, embedded in hierarchies of sets, is the mathematical theory of types and equivalence relations. This argument helps explain why the discrete sound units of phonology have been so useful in linguistics—because, like mathematics, they provide a tool for rigorous description. In this paper, we take a different approach from Peterson and Harary. Rather than using the language of mathematics to motivate phonemic theory, we use it to rigorously describe a model that provides an alternative to the linguistic representation of discrete sound units, at least for understanding spoken language production. Our immediate objective is to demonstrate that the hypothesis of whole word production is compatible with adult-like speech motor control, which references speech motor goals. The larger objective is to formalize a developmentally sensitive theory of production that limits the serial order problem in spoken language to the level of phrase production.

### 1.1. The Problem

The literate adult's awareness of discrete sounds in speech has motivated psycholinguistic theory as much as linguistic theory. Phonemes, in particular, have for a long while been understood as psychologically real units of language (Baudouin de Courtenay, 1881, cited in Koerner, 1972; Chomsky and Halle, 1965; Fromkin, 1971). One implication of this idea is that phonemes are relevant to speech production. In fact, a great deal of work in speech production since the 1970s has explicitly argued as much (e.g., Fromkin, 1971; Shattuck-Hufnagel, 1979; Stemberger, 1982; Dell, 1986; Levelt, 1989; Guenther, 1995; Roelofs, 1997; Schiller, 2000; Goldrick and Rapp, 2007; Hickok and Poeppel, 2007; Hickok, 2012; Turk and Shattuck-Hufnagel, 2014). A consequence of this hypothesis is the serial order problem (Lashley, 1951); that is, the problem of how discrete units are sequenced for output^1^. Psycholinguistic theory has addressed this problem by proposing a speech planning phase during production (see, e.g., Shattuck-Hufnagel, 1979; Levelt, 1989; Roelofs, 1997; Schiller, 2000; Goldrick and Rapp, 2007). This phase, known as phonological/phonetic encoding, is characterized as a sequential process of word form encoding that begins with phoneme sequencing within prosodic frames and ends with the context-dependent specification of phonetic information. Elsewhere, Redford has argued against this encoding hypothesis on developmental grounds (Redford, 2015, 2019); others have noted its incompatibility with the evidence that disruptions to phonological working memory do not in fact disrupt speech production any differently than, say, disruptions to visual-spatial working memory (Gathercole and Baddeley, 1993, p. Ch.4; Lee and Redford, 2015). Relatedly, whole research programs in phonetics and phonology (e.g., Autosegmental Phonology, Articulatory Phonology) have questioned the psychological reality of the phoneme and its importance in sequential speech planning based on evidence such as the long-distance acoustic and motor dependencies between “segments” in speech (i.e., coarticulation). Yet these programs also propose discrete phonological representations; for example, autosegmental phonologists favor distinctive features and articulatory phonologists propose the gesture, which is similar is some respects to the distinctive feature. Here, we argue against the general idea that discrete linguistic representations of sound are relevant to speech planning, and for the alternative, which is that word forms are remembered and retrieved holistically for production.

The whole word production hypothesis is particularly important and long-standing in child phonology where it has been used to explain the variability in a child's repeated production of the same word, the relationship between the child's production and the adult target, and the relationship between different words in the child's productive repertoire (Vihman and Croft, 2007; see also Vihman and Keren-Portnoy, 2013, and contributions therein). A version of the hypothesis is also advanced in Articulatory Phonology where word form representations are articulatory gestalts; more specifically, they are abstract and overlapping representations of discrete linguistic gestures used to produce the word (Browman and Goldstein, 1989, 1992; Goldstein et al., 2006)^2^. Yet another version of the hypothesis is proposed in Redford's (2015, 2019) developmentally sensitive theory of spoken language production. In this theory, the representations that guide adult speech are imagined as identical in kind to the holistic perceptual and motor phonological forms that underlie early child language. The perceptual representations posited are whole words derived from the ambient language, as in exemplar theories of phonology (Goldinger, 1998; Pierrehumbert, 2001; Hawkins, 2003; Johnson, 2006); the motor representations are abstracted with speech practice from sensorimotor experience, as in schema theories of action control (Schmidt, 1975; Norman and Shallice, 1986; Arbib, 1992; Cooper and Shallice, 2000). The proposal in Redford (2019) is that the whole word perceptual and motor phonological forms are co-activated during production and integrated via the perceptual-motor map. We take this proposal to be a strong version of the whole word production hypothesis and defend it here.

The proposal that holistic phonological representations provide the plans that guide adult word production requires defense because adults produce highly differentiated speech sounds. To do so, the speaker must consistently achieve specific vocal tract constrictions in rapid sequence. These constrictions suggest speech motor goals, defined as planned outcomes that are referenced in the control of speech movement. The suggestion of goals is strongly supported by the many natural and experimental demonstrations of motor equivalence (see Perrier and Fuchs, 2015). For example, adult speakers adapt nearly immediately to unexpected perturbations of the lips and jaw to achieve bilabial closure for bilabial consonants (Folkins and Zimmermann, 1982; Kelso et al., 1984; Shaiman and Gracco, 2002; van Lisehout and Neufeld, 2014); they also make very rapid adjustments during repeated productions of the same vowel if the auditory feedback they receive does not match the formant frequencies of the vowel they intended to produce (Houde and Jordan, 1998; MacDonald et al., 2010; Katseff et al., 2012; Lametti et al., 2012). The different types of adjustments indicate the importance of different types of information in speech motor control: the nearly instantaneous adaptation to mechanical perturbations of the articulators suggests that specific vocal tract constrictions are goals (e.g., Saltzman and Munhall, 1989; Liberman and Whalen, 2000; Sorensen and Gafos, 2016); on-going adjustment to articulation in response to perturbed auditory or sensory feedback suggests perceptual goals (e.g., Katseff et al., 2012; Lametti et al., 2012). But no matter the type of goals assumed, they are linked to discrete phonological representations in current theory. When the goal is a constriction, its phonological representation is the linguistic gesture; when it is perceptual, it is associated with the phoneme.

In this paper, we seek to accommodate the evidence for goals in speech motor control absent discrete phonological representations. More specifically, we address the challenge implicit in Bohland's (Bohland et al., 2010, p. 1509) argument against holistic phonological representations; namely, that the whole word production hypothesis “is incompatible with the exquisite control of vocal performance that speakers/singers retain for even the highest frequency syllables.” Our approach to this challenge is to model the integration of holistic perceptual and motor plans via the perceptual-motor map. The model we develop shares many assumptions of an information processing approach to speech motor control, especially the assumption that perception is important for speech motor control. The key difference is that our focus is not on the execution of speech, but rather on how perceptual-motor units of production emerge as motor space is reticulated with language acquisition. Another fundamental difference is that we explicitly address the relationship between phonology and speech motor control, and, in so doing, propose a motor phonological representation that is substantively different from the representations posited in current linguistic theory. Overall, the model objective is to demonstrate the in principle plausibility of the whole word hypothesis for understanding production in the context of adult-like speech articulation. Future research will address the in principle plausibility of the hypothesis for understanding production in the context of speech errors. This future work is necessary to complete the argument that the serial order problem in speech should be limited to sequencing words. We acknowledge that speech errors are a major source of evidence for a hypothetical phonological/phonetic encoding stage in speech production.

## 2. The Core Model

Perceptual-motor integration is a core assumption in neuropsychological models of speech production that assume perceptual goals (e.g., Guenther, 1995, 2006, 2016; Hickok and Poeppel, 2007; Houde and Nagarajan, 2011; Gow, 2012; Hickok, 2012). The Directions into Velocities of Articulators (DIVA; Guenther, 1995, 2006, 2016) is perhaps the best known and most completely developed of these models. DIVA provides a framework for understanding both the neuropsychology of speech motor control and the details of this control, including motor equivalence and coarticulation. In contrast, we seek to demonstrate that the whole word production hypothesis is compatible with adult-like speech motor control. To do this, we imagine perceptual-motor integration in speech from a developmental perspective given the domain knowledge of a phonetician. The result is the Core model, which is proposed here in the context of Redford (2015, 2019) developmentally sensitive theory of spoken language production.

The Core model is similar to DIVA in that it assumes a sound space and a motor space; it also envisions the perceptual-motor integration of speech with reference to trajectories through these spaces; however, the motor space in Core is more similar to the somatosensory space in DIVA than to its motor space. This is because Core does not address control over articulatory movements *per se*. The model is in fact agnostic on the question of how articulatory movements are themselves organized given a particular trajectory^3^. Another difference between the models is that, in Core, adult-like production relies by default on state feedback control rather than on feedforward processes (see Houde and Nagarajan, 2011). Thus, a matching and selection process on perceptual trajectories determines the path taken through motor space. Importantly, this process references holistic phonological representations that are the speech plan. By contrast, in DIVA, trajectories are defined by the sequential activation of cells in the speech sound map—that is, by a discretized plan. Below, we provide an informal overview of the Core model. This entails the introduction of a number of model specific terms. More precise definitions of these terms are given later when the model is more rigorously described.

### 2.1. Overview

**Figure 1:**
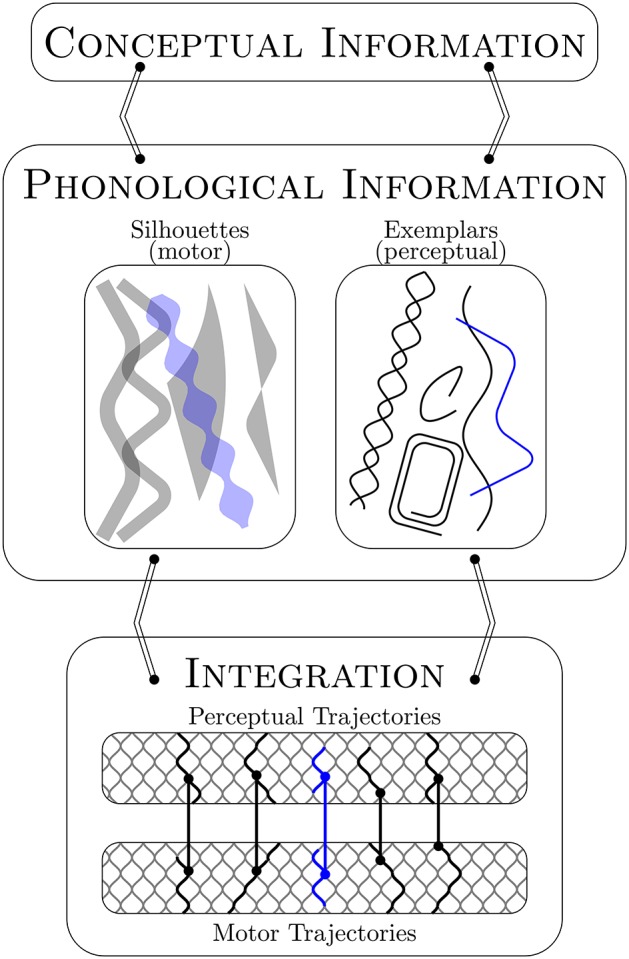
Core assumes distinct sets of holistic motor and perceptual forms as phonological representations (i.e., silhouettes and exemplars). These forms are co-activated in production via their shared conceptual information (blue). They are integrated for execution via the perceptual-motor map through a matching and selection process that is perceptually driven and motorically constrained. This process takes advantage of perceptual-motor units that arise through developmental time from the (near) overlap of motor trajectories in motor space. See text for details.

Core is designed to accommodate developmental change and the flow of activation in speech production from conceptualization to perceptual-motor integration. The proposed representations allow for change. The major components, or levels, in the model indicate flow in the production process. Figure 1 illustrates the relationship between the representations and levels to help frame the informal narrative description of the model given in this section.

Core assumes phonological representations that are distinct sets of holistic perceptual and motor forms associated with specific meanings: for example, with a nominal category like “dog,” a social-pragmatic category like “psst,” or a discourse device like “by the way.” The perceptual word forms are *exemplars*. The acquisition of these require that the listener segment ambient language input into meaningful units. The relevant input is speech produced by those with whom the listener interacts or to whom they otherwise attend, which is why the auditory memories are socially indexed (see Goldinger, 1998; Pierrehumbert, 2001; Hawkins, 2003; Johnson, 2006). The motor word forms are schema composites we call *silhouettes*. A *schema* is the memory trace of a motor pattern (= *motor trajectory* in Core) that a speaker has used to successfully communicate a specific meaning (i.e., a word). As with the more generalized schema proposed in Redford (2015), the notion of a silhouette proposed here takes inspiration from whole word approaches to child phonology (for a review see Vihman and Keren-Portnoy, 2013), information processing approaches to movement sequence learning and control (e.g., Klapp, 1975; Schmidt, 1975; Keele and Summers, 1976; Norman and Shallice, 1986; Arbib, 1992; Cooper and Shallice, 2006), and the early view of word form representations in Articulatory Phonology (see Browman and Goldstein, 1992). When one speaks, exemplars and silhouettes are integrated for execution via a perceptual-motor map. The map is not part of the linguistic system *per se* because it is initialized during the prelinguistic period (see also Guenther, 1995; Kuhl, 2000; MacNeilage and Davis, 2000; Hickok et al., 2003; Menn et al., 2013; Vihman, 2014). The map can therefore be accessed independently of meaning, for example, to mimic ambient noises^4^. In the Core model, the perceptual-motor map is the set of links between the motor and perceptual trajectories that wend through motor and perceptual spaces, respectively. These links are established with vocal-motor exploration. For every vocalization an infant produces, the trace of the motor pattern used in production is preserved as a motor trajectory that is linked at each point in time to the auditory memory of that vocalization, which is the perceptual trajectory. The motor space is simplified as the set of articulatory configurations, or possible vocal tract states, within a multidimensional articulatory space. The perceptual space is simplified as the set of possible sounds in a multidimensional acoustic space. The articulatory and acoustic dimensions structure the motor and perceptual spaces in such a way that articulatory and perceptual distances can be defined. These notions of distance are critical to a number of processes in Core. The notion of articulatory distance also provides the basis for a critical hypothesis that is instantiated in the Core model: when motor trajectories approach one another in motor space to the point of (near) crossing, junctures are created that can then be exploited to generate a new trajectory that is the combination of existing (partially) adjacent trajectories.

**Figure 2:**
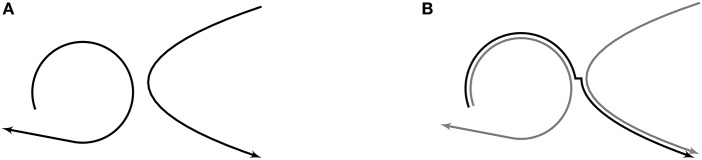
Junctures are created where trajectories near one another in motor space. This allows for the piece-wise combination of existing trajectories; for example, the trajectories associated with [bɑp] and [dɑg] (left) can be combined via the junctures that occur at [ɑ] to create a new trajectory, [bɑg] (right). Note that the curve representing the motor trajectory for [bɑg] is offset from where it truly is (on top of the other trajectories) so that all trajectories can be seen. For more information on how to read the illustrations, see section 2.4. **(A)** Representations of motor trajectories for [bχp] (left) and [dχg] (right). **(B)** Representations of motor trajectories for [bɑp] and [dɑg] in gray, and motor trajectory for [bɑg] in black.

The central idea behind the critical hypothesis is exemplified in Figure 2, which shows how the motor trajectories associated with [bɑp] and [dɑg] (left) can be used to produce [bɑg] (right) via the junctures created in motor space where the [ɑ] portion of the trajectories near one another. As this example makes clear, junctures index sets of (nearly) identical articulatory configurations. And, like all articulatory configurations along motor trajectories, the configurations at junctures are linked to sounds along corresponding trajectories in perceptual space. In this way, clusters of configurations at junctures in motor space, along with their corresponding perceptual points, can be considered the perceptual-motor units of speech. In Core, these units serve as pivots—places to transition from one motor trajectory to another along perceptually-linked paths that are selected to produce best approximations of whole word targets, as described below.

During the first word stage of language acquisition, an infant approximates a conceptually-linked exemplar drawn from the ambient language in the following way: the infant chooses an existing motor trajectory that is linked to a perceptual trajectory that is most similar to the exemplar being attempted. In this way, Core instantiates Vihman's (1993; 1996) hypothesis of first word production using vocal motor schemes: an infant's first words are based on familiar patterns from, say, babbling, that best approximate (perceptually) an adult target word (e.g., “ba” for “ball”). To account for developmental change beyond first words, Core assumes exemplars that are whole word forms^5^. These are represented as conceptually-linked perceptual trajectories that inhabit the same space as endogenous (i.e., self-generated) forms^6^. Similarity estimates between exogenous and endogenous perceptual trajectories are not necessarily based on the entire form. Instead, the estimates are biased toward matching the most salient aspects of the conceptually-linked trajectory, where salience is understood as subjective within certain acoustically defined bounds. Importantly, subjective salience is hypothesized to be governed by attention. What is salient during an attempt at matching any given exemplar can therefore change with experience. This change gives rise to the variable productions of early child language and, eventually, to adult-like productions of target words. So, for example, an infant might first try to match just the acoustically robust stressed syllable of a disyllabic word exemplar (e.g., “ba” for “bottle”)^7^. Having done so, perhaps repeatedly, the infant will likely find the less robust unstressed syllable relatively more salient and, in subsequent productions, may seek to also match its quantity and/or quality (e.g., “baba” for “bottle”)^8^. In this way, the assumptions of a non-linguistic basis for first word productions, holistic perceptual word form representations, and experience dependent changes in salience interact in the Core model to capture spoken language development. Successful communication during first word production triggers schema formation; that is, communicative success serves as the positive reinforcement needed to forge an associative link between a motor trajectory and lexical concept^9^. When the same concept is next selected for output, the newly established schema is activated along with the perceptual trajectory of the relevant exemplar. It is at this point that word production can be conceived of as the integration of perceptual and motor forms. Although the schema now biases production in the direction of the previously used motor trajectory, attention to different aspects of the co-activated exemplar will encourage some modification to or elaboration of the original motor trajectory. So, a second or third or fifth production of a single word is very likely to be different from the first. Each different successful production gives rise to a new schema, that is, to an additional motor trajectory with a link to the same lexical concept. These schemas are compiled to create a composite motor phonological form—the silhouette. This holistic representation then serves to define a swath through motor space during the integration process. This swath is narrow for those aspects of production that remain constant across many attempts at matching the exemplar, and wide elsewhere. Exemplar-driven exploration within and around this swath reticulates the motor space further, giving rise to additional junctures in areas of (near) articulatory overlap.

Key aspects of the Core model are formalized in the sections that follow. The formalization serves both to rigorously specify the interrelated hypotheses presented above and to demonstrate how these work together to yield perceptual-motor units absent their discrete specification in the phonology. The model presentation is organized developmentally, from infancy and prelinguistic vocalizations to early childhood and the emergence of an adult-like production process. We begin, though, with definitions of the perceptual-motor map and the acoustic and articulatory dimensions that structure the perceptual and motor spaces, respectively.

### 2.2. The Perceptual-Motor Map

**Figure 3:**
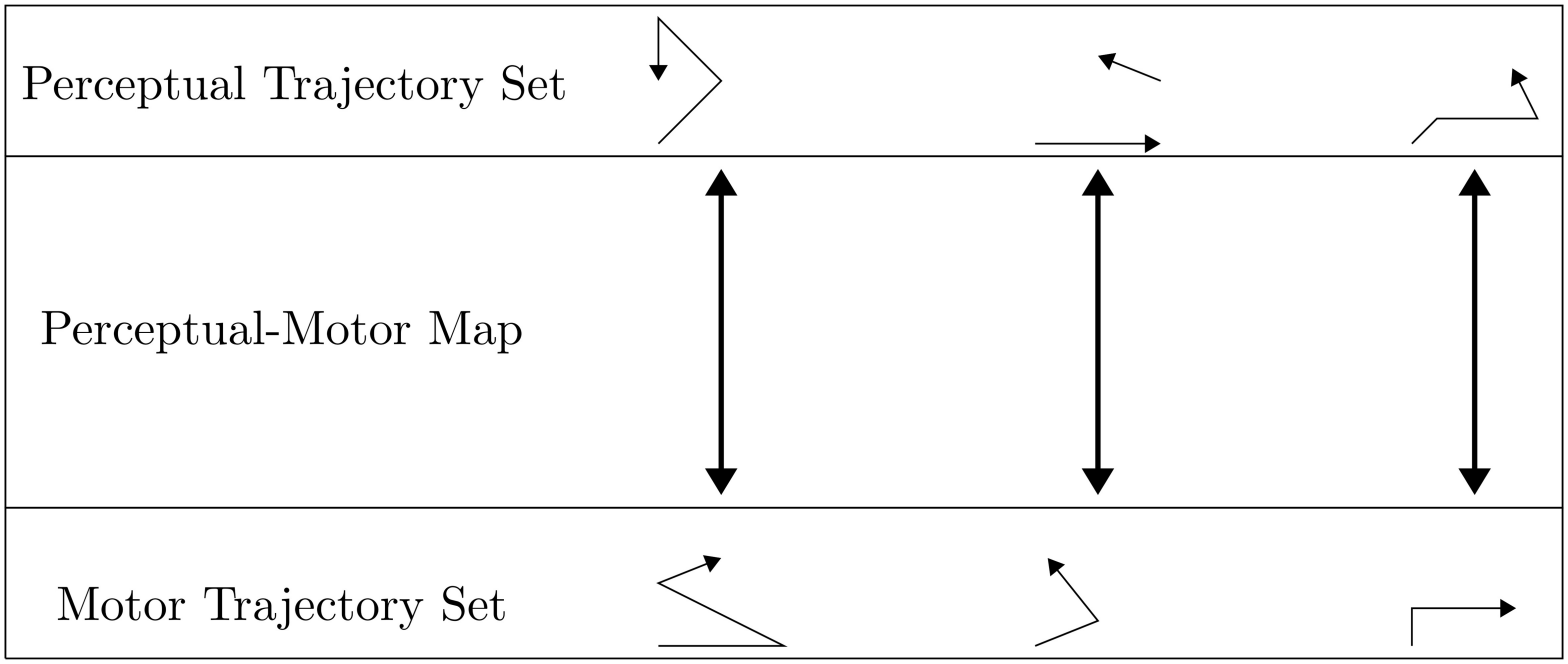
The perceptual-motor map is conceived of as a linked set of motor and perceptual trajectories. The trajectories themselves are drawn as arrowed paths, which are often modeled as not continuous in perceptual space due to stop closures and changes in periodicity among other factors.

Holistic perceptual and motor phonological representations are integrated for execution via the non-linguistic perceptual-motor map, which is defined as the set of links between paired trajectories that exist in perceptual and motor spaces, respectively. More specifically, the map is a bijection between the perceptual trajectory set and the motor trajectory set^10^, and so can be thought of as the set of bidirectional arrows between the sets of trajectories as shown in Figure 3. The initial set of links, or bidirectional arrows, is established during the prelinguistic period, as described in section 2.3. In this section, we rigorously define the perceptual and motor spaces, including the topologies of these spaces, and what we mean by trajectories through these spaces.

#### 2.2.1. Perceptual and Motor Spaces

The perceptual space is a set of points in Core, denoted sounds. Each point represents an “instantaneous” sound^11^, which is defined along the following 12 acoustic dimensions: the time derivative of loudness in phons, periodicity of the waveform, the first 3 Bark-transformed formants in the spectrum, the spectral center of gravity, the width of the spectral peak, and the time derivatives of each of these frequency dimensions^12^. It is possible that instantaneous sounds would be better represented with reference to the full speech spectrum (e.g., mel-frequency cepstrum), but the argument here does not depend on an exact representation of sound. Instead, the dimensions are illustrative and chosen with the goal of adequately and intuitively characterizing speech sounds for the phonetically informed reader. To complete this characterization, the dimensions are given the following values: periodicity is categorical and set to zero if the sound is aperiodic (e.g., voiceless fricative) and one if the sound is periodic (e.g., liquid); each of the other dimensions are set to some numerical value appropriate to the sound if the dimension is relevant for that sound, and set to zero otherwise. So, for example, when a sound is aperiodic, the Bark-transformed formant values (and their derivatives) are set to zero; when a sound is periodic, the center-of-gravity and width-of-peak values (and their derivatives) are set to zero to further distinguish sonorants from obstruents in perceptual space. Some nasals have an *F*_3_ value of zero; in this case, we set the Bark-transformed value to zero. Formally, then, an instantaneous sound is a 12-tuple:

$$\begin{array}{l} (\frac{d}{dt}(\text{LOUDNESS}),\text{PER},Z_{1},Z_{2},Z_{3},\text{COG},\text{WIDTH},\frac{d}{dt}(Z_{1}),\\ \;\frac{d}{dt}(Z_{2}),\frac{d}{dt}(Z_{3}),\frac{d}{dt}(\text{COG}),\frac{d}{dt}(\text{WIDTH})) \end{array}$$

where $\frac{d}{dt}\left(\text{LOUDNESS}\right)$ is equal to the time derivative of the phon value for the current sound; per = 1 if that current sound is periodic and per = 0 if it is aperiodic; *Z*_1_, *Z*_2_, and *Z*_3_ are equal to the first three Bark-transformed formant values if the sound is periodic and are equal to zero otherwise (with *Z*_3_ also being zero for certain nasals as described above); cog is equal to the spectral center of gravity if the sound is aperiodic, and is zero otherwise; width is equal to the width of the dominant spectral peak if the sound is aperiodic, and is zero otherwise; and where $\frac{d}{dt}\left(Z_{1}\right),\frac{d}{dt}\left(Z_{2}\right),\frac{d}{dt}\left(Z_{3}\right),\frac{d}{dt}\left(\text{COG}\right),\frac{d}{dt}\left(\text{WIDTH}\right)$ are equal to the time derivatives of the different spectral values^13^.

Although an instantaneous sound is mainly defined along dimensions that reference familiar acoustic measures of speech, the reference to time derivatives of acoustic properties is admittedly unusual and so requires explanation. In Core, an instantaneous sound is only ever realized as part of a trajectory. Derivatives allow us to code, at each point in time, the direction and extent of change along the intensity-related and spectral dimensions of this trajectory. This information is used to capture the amplitude and frequency modulation of the speech signal, which is critical for recovering place and manner of articulation information (e.g., Viswanathan et al., 2014). Including this as part of the representation of each point in the space ensures that if two trajectories (defined in section 2.2.3) pass through the same point in the space, they are perceptually equivalent at that moment. Note that our inclusion of dynamic information in the model assumes that infants also use such information when listening to speech. This assumption is reasonable based on the evidence that auditory temporal resolution is already adult-like by 6 months of age in typically developing infants (see Trainor et al., 2001).

Like the perceptual space, the motor space is a set of points in Core, denoted artic. In this case, the points represent all possible articulatory configurations for the speaker. These configurations describe the overall physical state of the vocal tract at any given moment in time during a vocalization; they are not goal states. Thus, artic, or the set of all possible articulatory configurations, can be used to represent continuous change in the vocal tract during production.

An articulatory configuration, and therefore the motor space, is defined along 20 dimensions: glottal width, 8 cross-sectional areas of the vocal tract, velum height, the time derivatives of each of the 8 cross-sectional areas and velum height, and the opening and closing phases of the jaw cycle. The cross-sectional areas of the vocal tract describe the result of coordinated actions, including laryngeal raising, pharyngeal constriction, and the movements of the tongue and lips with reference to the hard palate and maxilla (e.g., Fant, 1960)^14^. The specific choice of 8 segments is not critical to the model but is chosen here based on acoustic tube modeling work that considers consonantal articulation in addition to vowel articulation (Mrayati et al., 1988; Carré, 2004). Cross-sectional areas provide static information about jaw height given articulatory synergies between the jaw and tongue and between the jaw and lips; opening and closing phases of the jaw cycle are included as its own dimension in motor space in order to provide directional information, much like the time derivatives of acoustic properties in perceptual space. Such information is hypothesized to be relevant for delimiting syllable-sized articulatory timing relations (Redford, 1999; Redford et al., 2001; Redford and Miikkulainen, 2007), which will become important later. Formally, then, an articulatory configuration is the 20-tuple $\left(g,c_{1},c_{2},\ldots ,c_{8},v,\frac{d}{dt}\left(c_{1}\right),\frac{d}{dt}\left(c_{2}\right),\ldots ,\frac{d}{dt}\left(c_{8}\right),\frac{d}{dt}\left(v\right),j_{dir}\right)$ where *g* takes values in between 0 and 1 for glottal widths between fully closed (*g* = 0) and fully open (*g* = 1); *c*_*i*_ is the normalized cross-sectional area of the *i*th vocal tract segment, where *c*_*i*_ = 0 for a minimum area, and *c*_*i*_ = 1 for a maximum area; *v* = 0 when the velum is lowered, *v* = 1 when the velum is raised, and *v* takes some appropriate value between 0 and 1 when the velum is between lowered and raised; and *j*_*dir*_ takes a value between 0 and 1 during jaw opening, where *j*_*dir*_ = 1 when opening is executed with maximum force, *j*_*dir*_ takes a value between −1 and 0 during jaw closing, where *j*_*dir*_ = −1 when closing is executed with maximum force, and *j*_*dir*_ = 0 when the jaw is neither opening nor closing and so force is 0. Note that, for ease of some formal definitions, artic can be thought of as being embedded in a larger set – the set of all 20-tuples of real numbers; however, this larger theoretical set includes impossible configurations as well as the possible ones that make up artic.

We conclude this section with the following caveats. The focus in Core on sound and articulatory configurations for defining the perceptual and motor spaces is a simplifying choice. The dimensions we use to define these spaces are also simplified descriptions of acoustic and articulatory information. A more complete model would include additional dimensions and a sense of how these are weighted and normalized with respect to one another. It might also include, like DIVA, an additional layer in the map to solve the problems of articulatory coordination and timing that are not addressed here. Still, as defined, the dimensions in Core adequately describe human vocalzations, including word production. They also structure the perceptual and motor spaces in a manner that provides a formal foundation for the demonstration that perceptual-motor units of speech motor control can arise within the perceptual-motor map over developmental time absent discretized phonological input to the map.

#### 2.2.2. Perceptual and Articulatory Distance

The perceptual and motor spaces in Core are structured by the perceptual distance between instantaneous sounds and the articulatory distance between articulatory configurations. Defining the distance between every pair of points in motor space allows for the computation of distance between any two trajectories through motor space, which in turn allows for comparison of these trajectories; and similarly for perceptual space and perceptual trajectories. In Core, perceptual distance is relevant for word production and, later in development, for perceptually guided speech motor control (see Redford, 2019); that said, the argument in this paper is that the perceptual-motor units that arise with vocal exploration and spoken language acquisition are due to trajectory (near) overlap in motor space, not perceptual space. For this reason, we do not define a distance metric on the set of points in perceptual space, but assume that the distance between two instantaneous sounds should rely on some combination of the following values: differences between the corresponding coordinates except for *Z*_1_, *Z*_2_, and *Z*_3_, and the differences between the respective values of *Z*_3_−*Z*_1_ and the respective values of *Z*_3_−*Z*_2_ (these relative values are to normalize for physiological difference between speakers)^15^. Further, we assume an appropriate distance metric exists that is based on these variables.

Unlike perceptual distance, articulatory distance is central to the emergence of production units in Core and therefore to the argument of this paper. A specific distance metric, *d*_artic_, for articulatory distance is therefore proposed: the Euclidean distance metric on the set of articulatory configurations. Thus, for two articulatory configurations $a=\left(g,c_{1},\ldots ,c_{8},v,\frac{d}{dt}\left(c_{1}\right),\ldots ,\frac{d}{dt}\left(c_{8}\right),\frac{d}{dt}\left(v\right),j_{dir}\right)$ and $a^{\prime }=\left(g^{\prime },c_{1}^{\prime },\ldots ,c_{8}^{\prime },v^{\prime },\frac{d}{dt}\left(c_{1}^{\prime }\right),\ldots ,\frac{d}{dt}\left(c_{8}^{\prime }\right),\frac{d}{dt}\left(v^{\prime }\right),j_{dir}^{\prime }\right)$, the distance between the two is defined to be

$$\begin{array}{c} d_{\text{ARTIC}}(a,a^{\prime })\\ =\sqrt{\begin{array}{l} {\left(g-g^{\prime }\right)}^{2}+{\left(c_{1}-c_{1}^{\prime }\right)}^{2}+\ldots +{\left(c_{8}-c_{8}^{\prime }\right)}^{2}+{\left(v-v^{\prime }\right)}^{2}\\ \;+{\left(\frac{d}{dt}(c_{1}-c_{1}^{\prime })\right)}^{2}+\ldots +{\left(\frac{d}{dt}(c_{8}-c_{8}^{\prime })\right)}^{2}\\ \;+{\left(\frac{d}{dt}(v-v^{\prime })\right)}^{2}+{\left(j_{dir}-j_{dir}^{\prime }\right)}^{2}. \end{array}} \end{array}$$

Note that if we were to define *d*_artic_ in almost the same way, but using only the variables for glottal width, cross-sectional vocal tract areas, and velum openness, the distance between two articulatory configurations would match a phonetician's intuition of articulatory distance. Differences between jaw direction values are included to capture the additional intuition that achieving a particular vocal tract configuration while opening the mouth is different than achieving the same configuration while in the process of closing the mouth (see, e.g., Fujimura, 1990). Recall that jaw direction also allows us to define syllable-sized articulatory timing relations (Redford, 1999; Redford et al., 2001; Redford and Miikkulainen, 2007).

In addition to structuring the perceptual and motor spaces, the notions of perceptual and articulatory distances allow for the comparison of trajectories in these spaces. In Core, comparisons between perceptual trajectories are fundamental to the production of first words, comparisons between motor trajectories are fundamental to the evolution of motor representations, and comparisons of linked pairs of trajectories to targeted perceptual and motor representations are fundamental to the integration of these forms during production. Since two of these processes force further reticulation of motor space over developmental time, comparisons are also fundamental to the emergence of junctures. Junctures enable novel word generation in Core and the development of adult-like speech motor control.

#### 2.2.3. Perceptual and Motor Trajectories

Perceptual and motor trajectories are defined as functions from time intervals to perceptual space and motor space, respectively. A perceptual trajectory takes time as an input and gives as an output the instantaneous sound at each time; a motor trajectory also takes time as an input, and gives as an output the articulatory configuration at each time.

The mathematical structure imposed on motor space by the distance metric *d*_artic_ organizes articulatory configurations so that the structure is consistent with intuitive notions about continuous physical motion. More specifically, the articulatory distance metric defined in section 2.2.2 induces a topology on motor space. Assuming the standard metric-induced topology on real intervals (i.e., the domains of motor trajectories), the continuity of motor trajectories can be assessed with reference to the structured motor space. In Core, we claim that every motor trajectory is a continuous function according to these topologies. This is a critical claim for the procedures defined below and, of course, also coincides with the facts of speech: in order to go from one articulatory configuration to another, the vocal tract must go through intermediate states such that each of our variables changes continuously; for example, in order for the 5th segment of the vocal tract to go from having a cross-sectional area of 3 to 1 cm^2^, it must go through stages in which it attains cross-sectional areas of 2, 1.5, 1.124 cm^2^, and so on. Put another way, since the notion of distance defined herein aligns with the reality of articulation, the notion of continuity as rigorously defined aligns with the reality of continuous motion.

Although functions of time, trajectories code only relative time. To normalize for absolute time, we define equivalence relations. In motor space, two trajectories are equivalent if one can be uniformly temporally stretched to create the other. Specifically, two motor trajectories *m*:[0, *T*] → artic and *n*:[0, *U*] → artic (i.e., motor trajectories with domains [0, *T*] and [0, *U*], respectively) are equivalent if and only if $m\left(t\right)=n\left(\frac{U}{T}t\right)$ everywhere on their domains^16^. This equivalence relation yields a set of equivalence classes of trajectories. In Core, every equivalence class yields a representative motor trajectory that has the domain [0, *s*], where *s* is the number of syllables for each motor trajectory within that class. The value of *s* is well-defined because syllable number is determined by *j*_*dir*_ and so is the same for all motor trajectories within a single class. The representative (time normalized) motor trajectory is the one used in the production processes described below.

An analogous equivalence relation is imposed on the set of perceptual trajectories. Thus, if two motor trajectories are equivalent, then their perceptual counterparts will also be equivalent. In this way, the equivalence relation imposed on motor space is also a property of the perceptual-motor map. Note, however, that we are not able to as easily choose a representative of each perceptual equivalence class because syllable information, derived from *j*_*dir*_, is only available for perceptual trajectories that are already linked to motor trajectories (i.e., self-productions). Exemplars, which inhabit the same space as self-productions, have no associated motor trajectories and so no syllable information. When syllable number is available for the perceptual trajectories, they are normalized using this information; otherwise, they are normalized using an arbitrary domain length, since the processes themselves implicitly normalize for domain length.

### 2.3. Initializing the Perceptual-Motor Map

Having defined the perceptual and motor spaces, a notion of distance in each space, trajectories through the spaces, and a procedure for time normalization, we turn now to the initialization of the perceptual-motor map.

Core embodies the familiar hypothesis that an infant's prelinguistic vocalizations give rise to the perceptual-motor map (Stark, 1986; Guenther, 1995; Kuhl, 2000; MacNeilage and Davis, 2000; Hickok et al., 2003; Menn et al., 2013). Here, an infant's prelinguistic vocalizations are specifically understood as developmentally constrained explorations of the vocal motor and acoustic perceptual spaces. We suppose that with an infant's every vocalization the parallel motor and perceptual spaces are explored and the links between them defined, giving rise to the perceptual-motor map. Specifically, each vocalization results in a motor memory trace and an auditory memory trace that are associated in time. Through this association, the transient traces become fixed and linked. These links are the set of paired motor and perceptual trajectories that constitute the perceptual-motor map. Motor and perceptual trajectories and a link between them are established with every vocalization, from infancy to adulthood.

The perceptual-motor map is initialized at birth with the infant's cries and vegetative sounds. As an infant gains voluntary control over laryngeal and other articulatory movements at around 8 weeks of age, the perceptual and motor spaces are more deliberately explored. Although the squeals, coos, raspberries, and so on that are produced during the phonatory and expansion stages grow the set of links that constitute the perceptual-motor map, we follow the lead of others and focus on babbling due to its importance in theories of speech acquisition (see, e.g., Oller, 1980; Guenther, 1995; MacNeilage and Davis, 2000). The repetitive nature of babbled utterances also makes them useful for formally introducing the Core concept of junctures, which is central to the acquisition of spoken language: as previously described, junctures give rise to perceptual-motor units; they also delimit smaller paths, or articulatory chunks, within larger trajectories that can then be combined to produce new vocalizations. The combination process becomes the focus of description in what follows below.

### 2.4. Junctures, Clusters, and Articulatory Chunks

The illustrations in Figure 2 convey the idea that junctures are created when motor trajectories approach one another in motor space. Junctures form in clusters with spoken language acquisition. These clusters, along with their corresponding (linked) perceptual points, represent perceptual-motor units of production at the level of speech motor control.

Junctures and clusters are defined based on trajectories in motor space—even though, as stated, the perceptual-motor units themselves entail the corresponding perceptual points. When a new motor trajectory *m* is created out of motor trajectories *k*_1_, …, *k*_ℓ_ as described in Appendix A, *k*_1_(β_1_), *k*_2_(α_2_), *k*_2_(β_2_), *k*_3_(α_3_), *k*_3_(β_3_), …, *k*_ℓ−1_(α_ℓ−1_), *k*_ℓ−1_(β_ℓ−1_), *k*_ℓ_(α_ℓ_) become junctures; that is, the endpoints of the small segments that connect existing trajectories to create a novel one all become junctures. Then, at any given moment in developmental time, a single-linkage hierarchical clustering process is applied to the set of junctures, where the process is stopped just before the height of the tree meets or exceeds ε, where ε is the parameter defined in Appendix A. As a developmental process, this clustering can be described as follows. When an articulatory configuration *a* becomes a juncture, there are three possibilities: (1) it could be “sufficiently close” to exactly one existing juncture point, where “sufficiently close” in this case means being a distance of less than ε away, where ε is a pre-defined parameter used in the process defined in Appendix A; (2) it could be “sufficiently close” to multiple existing junctures; or (3) it could have a distance of greater than or equal to ε from all existing juncture (i.e., not sufficiently close to any existing junctures). If *a* is less than ε away from a single juncture point (possibility 1), then *a* joins the cluster that juncture point belongs to. If *a* is less than ε away from more than one juncture point (possibility 2)—for example, *a* is less than ε from *a*_1_, …, *a*_*n*_, then the clusters that *a*_1_, …, *a*_*n*_ belong to merge into one cluster that also now includes *a*—that is, they merge via their mutual connection to *a*. If *a* is not within ε of any existing juncture point (possibility 3), the set {*a*} becomes its own cluster.

Note that a single novel production can trigger the establishment of multiple juncture points. Regardless of the order in which these juncture points are “added,” the process above yields the same clusters.

**Figure 4:**
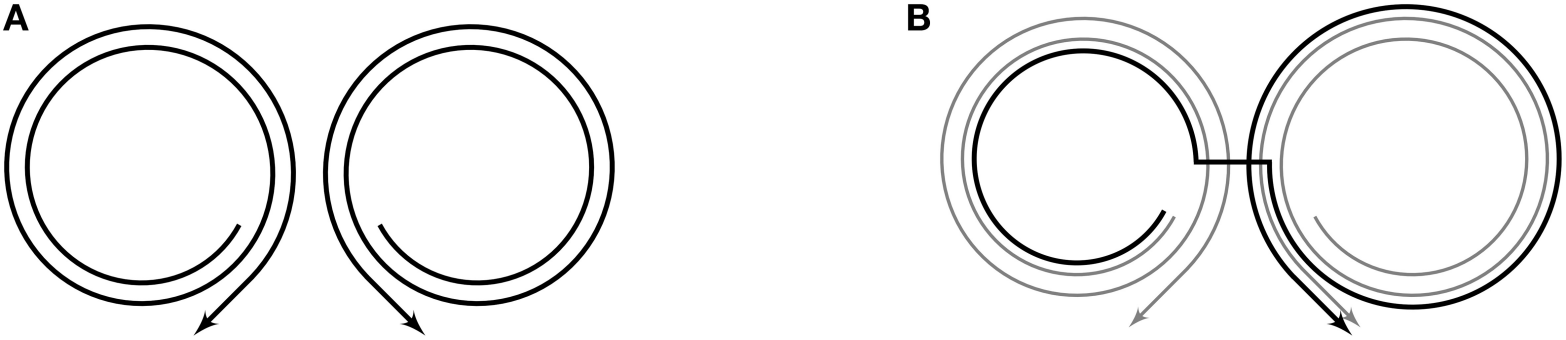
The juncture-delimited chunks within adjacent [bɑbɑ] and [dɑdɑ] trajectories are combined to produce the new trajectory/vocalization [bɑdɑ]. **(A)** Paths of motor trajectories for prespeech vocalizations [bɑbɑ] (left) and [dɑdɑ] (right). **(B)** The path for [bɑdɑ] in black, offset from its true path so that all trajectories are visible.

The early language function of junctures is to index locations where the speaker can deviate from one existing motor trajectory to pursue another. Since the juncture-delimited paths along existing trajectories are available to participate in novel trajectories through combinations, they can be thought of as articulatory chunks from which new utterances (e.g., words) can be built. The articulatory chunks are large in early development and small later on when many more junctures have arisen through exploration of the motor space. To illustrate chunking, we use figures in which the space on the page is treated as analogous to motor space, and where trajectories are represented as curves with direction through this space. Note that timing is *not* represented in the figures. For example, Figure 4 shows the junctures at the [ɑ] portions of the chunks [bɑbɑ] (left) and [dɑdɑ] (right). Junctures effectively delimit the chunks [bɑ] and [ɑdɑ], and make possible the combination [bɑdɑ]. In the remainder of this section, we formally describe the combinatorial process in Core with reference to the case of [bɑdɑ], beginning with the assumption that the articulatory configuration at the center of the first vowel in [bɑbɑ] is close enough in motor space to the articulatory configuration achieved at the center of the first vowel in [dɑdɑ] for a juncture to be created on each trajectory.

Let us first formally represent the motor trajectories for [bɑbɑ] and [dɑdɑ]. There are many motor trajectories that could accurately be described as yielding [bɑbɑ] and [dɑdɑ]. We choose two specific ones, *m*_1_ and *m*_2_, to build [bɑdɑ]. Most details of *m*_1_ and *m*_2_ are not relevant to the process, and so will be unspecified; what is relevant is the domain of these functions and the formal analog of the “close enough” assumption noted above. More specifically, both trajectories are two syllables so both have a domain of [0, 2]. So, we have *m*_1_:[0, 2] → artic as the motor trajectory for [bɑbɑ] and *m*_2_:[0, 2] → artic as the motor trajectory for [dɑdɑ]. Let *a*_1_ be the articulatory configuration achieved at the center of the first vowel in [bɑbɑ] and *a*_2_ that for [dɑdɑ]. For the sake of specificity, let the configurations occur at relative times 0.6 and 0.7 in their respective trajectories (the particular values are not central for the argument). This means that *m*_1_(0.6) = *a*_1_ and *m*_2_(0.7) = *a*_2_. Critically, our assumption is that *d*_artic_(*a*_1_, *a*_2_) is “sufficiently small” for there to be a juncture created at the endpoints of the segment from *m*_1_ to *m*_2_ going from *a*_1_ to *a*_2_; in the language of Appendix A, we assume that *d*_artic_(*a*_1_, *a*_2_) is smaller than the parameter ε – that is, we assume that criterion (*) is fulfilled. Then, the speaker can traverse the first part of the [bɑbɑ] trajectory and, once they reach articulatory configuration *a*_1_, make the small shift over to articulatory configuration *a*_2_ to follow along the rest of the [dɑdɑ] trajectory. Formally, making simplifying choices for a few of the parameters in Appendix A, we can define *m*:[0, 2] → artic by

$$m(t)=\{\begin{array}{ll} m_{1}(t) & 0\leq t<0.6\\ (1-\lambda (t))a_{1}+\lambda (t)a_{2} & 0.6\leq t<0.7\\ m_{2}(t) & 0.7\leq t\leq 2 \end{array}$$

where λ(*t*) = 10*t* − 6.

Even without referencing the specifics of Appendix A, one can see that *m* has been defined as the concatenation of a piece of *m*_1_ (that ends at vocal tract configuration *a*_1_), a connecting segment between *a*_1_ and *a*_2_, and a piece of *m*_2_ (that begins at vocal tract configuration *a*_2_). This clearly aligns with the illustration of this new trajectory shown in Figure 4.

More specifically, in reference to the variables in Appendix A, *s* = 2 (since the number of syllables in the resulting trajectory is 2), and, for simplicity of the formula above, we assume that δ_1_ = 0.1 (this is the normalized length of time it takes to shift from *m*_1_ to *m*_2_); these values together mean that *u* = 1 (this is a stretching parameter that ensures that the resulting trajectory, *m*, has the desirable domain). As stated above, we assumed that these trajectories were eligible for combination in the first place by assuming that *d*_artic_(*a*_1_, *a*_2_) was sufficiently small (in Appendix A, below the threshold value ε)^17^.

To summarize, the perceptual-motor exploration that occurs during the prelinguistic period initializes the perceptual-motor map with linked pairs of perceptual and motor trajectories. These can then be exploited to create new utterances via junctures at points of (near) overlap in motor space. The smaller paths delimited by junctures are articulatory chunks. The structure of these chunks is defined by the structure of prelinguistic vocalizations. For example, the repetitive nature of babbling is likely to result in chunks that are the size of syllables or demi-syllables, as suggested by the case considered above. In the next section, we turn to the onset of spoken language production when an infant begins to use articulatory chunks to produce first words. Keep in mind, though, that babbling continues alongside word production until about 18 months of age (Locke, 1989; Oller, 2000; Vihman, 2014). This means that the infant will continue to explore perceptual and motor spaces and will therefore continue to lay down entirely new trajectories through motor space while also building up initial motor phonological representations.

### 2.5. Perceptual-Motor Integration

In Redford's (2019) developmentally sensitive theory, adult speech production is imagined as the integration of holistic perceptual and motor phonological forms. Motor forms emerge from speech practice; perceptual forms are acquired. The acquisition of perceptual forms, or exemplars, depends both on the development of speech segmentation strategies and on the infant's insight that adult vocalizations convey conceptual information. Both of these conditions may be met as early as 7 months of age (Harris et al., 1995; Bergelson and Swingley, 2012). Let us assume then that it is at this point that the infant begins to acquire exemplars from the ambient language. Motor forms begin to emerge later, at around 12 months of age, with first word production. The production of first words is imagined in the theory as the moment when the infant, motivated to communicate a specific concept, selects a motor trajectory whose corresponding perceptual trajectory approximates the exemplar associated with that concept. In Core, this trajectory can be familiar (e.g., [bɑbɑ]) or novel (e.g., [bɑdɑ]). The important thing is that the trajectory be similar in some respects to the exemplar. This means that first word production requires a matching and selection process.

If the matching and selection process is successful, the infant will have communicated the intended concept to their interlocutor and induced some kind of desired response. In this case, the motor trace of the vocalization will be remembered in association with the concept that was communicated. This is the schema. In future attempts to communicate the same concept, the schema and exemplar associated with that concept are co-activated, biasing the matching-selection process in the direction of the previously used motor pattern. Still, the exemplar-matching objective of speech production remains. Thus, future attempts at the same word are likely to result in the selection of a new motor trajectory, especially if the infant attends to different aspects of the word during production (i.e., salience shifts). Each of these new selections, when they result in successful communication, generate new schemas. In Core, these are aligned and combined with the existing schema to define the silhouette—the motor phonological representation that evolves with developmental time.

Below, we rigorously describe the critical matching and selection process used in first word production, the motor phonological representations that result from this process, and the perceptual-motor integration process that characterizes production once motor phonological forms have been acquired.

#### 2.5.1. Matching and Selection

Recall that the perceptual space is denoted by sounds, and the distance metric on the perceptual space is denoted by *d*_sounds_. When an infant first attempts to communicate a concept, *c*, they choose a corresponding exemplar, *e*_*c*_, which becomes the perceptual goal for production. We claim that the goal is a function *e*_*c*_:[0, *T*] → sounds and is a perceptual trajectory that is not attached to a motor trajectory. Different portions of the exemplar will have different levels of salience to the infant^18^. Salience is described by the function salience_*e*_*c*__:[0, *T*] → [0, 1], which takes a time as an input, and gives as an output the salience of the sound that occurs at that time in the exemplar, where 1 indicates maximum salience and 0 indicates no salience. For example, suppose that the exemplar *e*_*c*_:[0, *T*] → sounds is two syllables and these syllables are of equal duration; suppose also that the first syllable—that is, the first temporal half of the trajectory (up to and including the midpoint)—is maximally salient to the infant and the second syllable—that is, the second temporal half of the trajectory—is not at all salient to the infant; then the salience function for *e*_*c*_ would be defined by

$$\text{SALIENC}{\text{E}}_{e_{c}}(t)=\{\begin{array}{cc} 1 & \text{if }0\leq t\leq \frac{1}{2}T\\ 0 & \text{if }\frac{1}{2}T<t\leq T \end{array}$$

Once salience is taken into account, the search begins to find a pair of corresponding perceptual and motor trajectories, *p*:[0, *s*] → sounds and *m*:[0, *s*] → artic, that best fulfill the criteria enumerated below. Note that we do not specify what it means to “best fulfill” these criteria, or relatedly, how the process of finding these optimal trajectories is executed; a few possibilities are nonetheless mentioned in the discussion.

1. *m* is an allowed (possibly novel) motor trajectory as described in section 2.2.3.
2. A particular weighted distance, as defined below, between *e*_*c*_ and *p* is small enough, where the weighting serves to make the distances on more salient parts of the exemplar more important than the distances on the less salient parts of the exemplar. More specifically, the weighted distance
  $$\int_{0}^{1}{\text{SALIENCE}}_{e_{c}}\left(Tt\right)d_{\text{SOUNDS}}\left(e_{c}\left(Tt\right),p\left(st\right)\right)dt$$
  is small enough.
  This expression first stretches *e*_*c*_ and *p* (by multiplying their inputs by *T* and *s*, respectively) so that the starting point of *e*_*c*_ is aligned with the starting point of *p*, and these both occur at *t* = 0, and the ending point of *e*_*c*_ is aligned with the ending point of *p*, and these both occur at *t* = 1. Indeed, observe that *e*_*c*_(*T* · 0) = *e*_*c*_(0) and *p*(*s* · 0) = *p*(0), which are the values these trajectories take initially, time-wise; and *e*_*c*_(*T* · 1) = *e*_*c*_(*T*) and *p*(*s* · 1) = *p*(*s*), which are the values these trajectories take finally, time-wise. Once these trajectories are properly aligned, the distance is computed between them for every value of *t*, and for each of these *t* values, multiplied by the salience of the exemplar at that time (where the salience function has also been stretched for alignment). Then, the average of all of these salience-weighted distances is computed. The result is the expression above. Thus, the smaller this expression, the better an approximation *p* is of *e*_*c*_. Note that this weighted distance is very similar to the “class of metrics” described by Mermelstein (Mermelstein, 1976, p. 96), the “time-normalized difference definition” given by Sakoe and Seibi (1978), and the approach is similar to that taken by Itakura (1975), among others.
3. *m* is a favored motor trajectory, where the notion of favored corresponds with frequency such that a trajectory is more favored if it has been traversed often, and a combination of motor trajectories is more favored if its constituent paths have been traversed together with one another often. Note that frequency is a relative value, and no claim is made here about the specific relationship between favoredness and frequency. The only claim is that favoredness increases as frequency increases.

Overall, then, the matching and selection process instantiated in Core is based on a perceptual approximation of the holistic goal, which is nonetheless constrained by existing paths through motor space. The approximation is further biased by the frequency with which the motor paths have been practiced together. The process therefore ensures that first words resemble patterns that have been most extensively practiced during vocal-motor exploration. In this way, Core accounts for the observation that children's favored forms in first words reflect their favored production patterns in babbling (Vihman et al., 1986; McCune and Vihman, 2001; c.f. Davis et al., 2002) and the observation that children tend to favor a limited number of forms in first word productions, even while what is favored differs across individual children (Ferguson and Farwell, 1975; Macken and Ferguson, 1981; Stoel-Gammon and Cooper, 1984; inter alia).

#### 2.5.2. Motor Representations and Convergence

The matching and selection process described so far considers the role of holistic perceptual phonological representations (i.e., exemplars) in production. In this section, we define holistic motor phonological representations (i.e., schemas and silhouettes) and formally describe their role in the production process.

In a first attempt at communicating a concept *c*, the motor trajectory *m* is selected for output. Given the very slow development of peripheral motor control (Smith and Zelaznik, 2004), the trajectory that is executed will be close to *m* but not exactly the same as *m*; that is, it will be *m* plus whatever noise is introduced during implementation. Let us call this new trajectory *m*′. If the vocalization from which *m*′ is derived successfully communicates *c*, then *m*′ will be linked to *c*. This is a schema, which we will refer to as ${\text{SCHEMA}}_{m^{\prime }}$. Note that ${\text{SCHEMA}}_{m^{\prime }}$ is the same function as *m*′, and only differs from *m*′ in that it is associated with the concept *c*. Once *c* has been successfully communicated using *m*′:[0, *s*′] → artic, and assuming all the objects used were those that were identified in the selection process described above (section 2.5.1), the next attempt to communicate *c* is as follows.

${\text{SCHEMA}}_{m^{\prime }}$ is activated at the same time as an exemplar associated with *c*. The specific exemplar may be different than before, so let us call it $e_{c}^{\prime }$. There is also a function ${\text{SALIENCE}}_{e_{c}^{\prime }}$. A pair of perceptual and motor trajectories, *m*_1_:[0, *s*_1_] → artic and *p*_1_:[0, *s*_1_] → sounds, is then chosen based on criteria 1-3 above (but with the appropriate objects substituted) and based on a fourth criterion:

4. *m*_1_ is close to the motor schema ${\text{SCHEMA}}_{m^{\prime }}$. More specifically, letting *k* be a fixed value, there exist α, β, with 0 ≤ α ≤ β−*k* ≤ *s*_1_−*k* such that
  $$\frac{1}{\beta -\alpha }\int_{\alpha }^{\beta }\left(d_{\text{ARTIC}}\left(m_{1}\left(t\right),m^{\prime }\left(h_{\alpha ,\beta ,s^{\prime }}\left(t\right)\right)\right)\right)\;dt$$
  is sufficiently small, where $h_{\alpha ,\beta ,s^{\prime }}\left(t\right)=\frac{s^{\prime }}{\beta -\alpha }t-\frac{s^{\prime }\alpha }{\beta -\alpha }$—this function is used to align *m*′ with the portion of *m*_1_ going from relative time α to relative time β. Recall that ${\text{SCHEMA}}_{m^{\prime }}$ is the same as *m*′, but with a link to a concept—so comparing something in motor space to ${\text{SCHEMA}}_{m^{\prime }}$ is the same as comparing it to *m*′. The value of *k* is the minimum length of a portion of *m*_1_ we are willing to have ${\text{SCHEMA}}_{m^{\prime }}$ align with.

**Figure 5:**
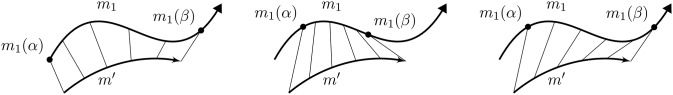
Three possible alignments of an existing schema trajectory (the lower curve in each picture) with a motor trajectory selected for output (the upper curve in each picture). In this figure, a constant velocity is assumed, which means that time is proportional to distance. In the first case (left), the existing schema trajectory is being compared with about the first 80% of the selected motor trajectory. In the other cases, it is being compared with some middle portion of the selected trajectory. Out of the three cases, the first alignment gives the smallest distance. It would be possible, for instance, that only the first alignment fulfills the criteria of the expression above being “small enough”. In that case, as long as β − α ≥ *k*, the criteria would considered fulfilled by *m*_1_, since there would *exist* a pair of values for α and β (i.e., an alignment) that makes this distance sufficiently small.

Regarding criterion 4 above, it is not required that the chosen motor trajectory and the schema be similar start to finish; for example, the new motor trajectory might have an additional syllable than the motor trajectory associated with the previous schema. So, instead of requiring all of ${\text{SCHEMA}}_{m^{\prime }}$ to match *m*_1_ well enough from start to finish—i.e., from times 0 to *s*_1_—the whole of ${\text{SCHEMA}}_{m^{\prime }}$ is allowed to be compared to *m*_1_ from times α to β, for various values of α and β, as shown in Figure 5. For any particular choice of α and β, *m*′ is temporally stretched (via the precomposition with $h_{\alpha ,\beta ,s^{\prime }}$) to run from α to β with respect to *m*_1_. Then, with this alignment, the average distance between the schema and the motor trajectory is computed. The expression above gives this average.

To avoid compressing ${\text{SCHEMA}}_{m^{\prime }}$ too much relative to *m*_1_, we specify that ${\text{SCHEMA}}_{m^{\prime }}$ cannot be compared to a portion of *m*_1_ that is less than *k* units of relative time, for some predetermined value of *k*. In other words, it must be the case that β − α ≥ *k*. Of course, it is also the case that α and β must be between 0 and *s*_1_, since they must be in the domain of *m*_1_. Combining these facts with the inequality β − α ≥ *k*, we get the chain of inequalities stated above, 0 ≤ α ≤ β − *k* ≤ *s*_1_ − *k*. Additionally, to retain the relative timing of ${\text{SCHEMA}}_{m^{\prime }}$, we only allow the temporal stretching to be linear—that is, by only allowing precomposition of *m*′ with a linear function, the only thing altered is which portion of *m*_1_ that *m*′ is being compared to—but within that comparison, the relative timing of *m*′ is maintained.

So, as stated above, the statement that *m*_1_ is close to the motor schema ${\text{SCHEMA}}_{m^{\prime }}$ just means that there are *some* α and β, with 0 ≤ α ≤ β − *k* ≤ *s*_1_ − *k*, such that the expression above—i.e., the average distance after alignment based on α and β—is sufficiently small.

In summary, a linked pair of perceptual and motor trajectories, *p*_1_:[0, *s*_1_] → sounds and *m*_1_:[0, *s*_1_] → artic, is selected for output based on some combination of how well the perceptual trajectory matches the perceptual goal (criterion 2), the extent to which the associated motor trajectory is favored (criterion 3), and the extent to which that motor trajectory matches the activated schema (criterion 4). Also, the motor trajectory must be achievable (criterion 1). When the matching and selection process references exemplars *and* schemas, speech production can be characterized as the perceptual-motor integration of holistic perceptual and motor phonological forms. Note however that the process in Core is not integration *per se*; instead, perceptual-motor integration is the convergence of a linked pair of trajectories that best approximate the perceptual goal within the constraints of past speech motor practice.

Whereas it is common to assume strong motor constraints on production in early child language (e.g., Locke, 1983; McCune and Vihman, 1987; Davis et al., 2002), it is also clear that these constraints are relaxed in adult language with the development of adult-like speech motor control. There are many sources of evidence for this assertion, including results from auditory feedback perturbation studies (e.g., MacDonald et al., 2010; Katseff et al., 2012) and phonetic imitation studies (e.g., Shockley et al., 2004; Nielsen, 2011; Babel, 2012). All together, the evidence strongly suggests that adult speech is perceptually guided, at least within the limits of the perceptual and motor spaces explored in one's native language [see, e.g., the limits of VOT imitation in (Nielsen's, 2011) study]. In Core, the transition from strong motor constraints on production to adult-like perceptually guided speech production results from the evolution of motor phonological representations through time (see also Redford, 2015, 2019). Let us consider this evolution next.

As with the first successful attempt at a word, subsequent successful attempts at the word yield new and different schemas. This is both because a child's attention to exemplar attributes changes through time (see discussion of “salience” in section 2.1) and because their immature motor systems introduce noise into the production process such that the motor space adjacent to a trajectory that has been selected for output is randomly explored. In Core, the new schemas generated with each successful new production of a word are associated with the target concept. All schemas associated with a single concept come together to form a silhouette, which we define recursively to emphasize our developmental perspective. To keep track of the silhouette's shape at any point in developmental time, we write sil_*c,n*_ to denote the silhouette that corresponds to *c* after the *n*th successful attempt to communicate *c*. When the moment in developmental time is not important, we will simply write sil_*c*_ to denote the silhouette corresponding to *c*, where the iteration is implicit. Then, to build the silhouette, the schemas are temporally aligned and the convex hull taken at each point in time of the outputs of the schemas^19^. The silhouette is defined to be a function that takes time as an input and gives the motorically possible subset of the convex hull corresponding to that time as an output; in other words, the silhouette encodes a time varying region. Note that the way we define a silhouette at each point in time uses a procedure similar to Guenther's (1995) convex region theory. Critically, though, DIVA's time varying regions contain exactly the vocalizations that are acceptable adult productions of a given speech sound. In contrast, a silhouette highlights a swath through motor space in Core; reference to a perceptual trajectory is required to find a good motor trajectory within the swath, namely, one that will yield an acceptable adult sound/word production.

Formally, the silhouette that is associated with *c* after *n* iterations will be a function ${\text{SIL}}_{c,n}:\left[0,s_{n}\right]\to P\left(\text{ARTIC}\right)$, where P(ARTIC) is the power set of the set artic (i.e., the set of all subsets of artic), and *s*_*n*_ is some number representing the number of syllables in sil_*c,n*_, and is derived from the constituent schemas and how these are aligned^20^. Although a silhouette, in the sense of a *composite* motor form, only really emerges after two different attempts at a word, here we consider the first silhouette to emerge after the first attempt at a word. So, suppose that the first schema for *c* is schema_*m*_1__:[0, *s*_1_] → artic; then the first silhouette, ${\text{SIL}}_{c,1}:\left[0,s_{1}\right]\to P\left(\text{ARTIC}\right)$ is defined by sil_*c*, 1_(*t*) = {schema_*m*_1__(*t*)}. This defines the silhouette as nearly the same function as schema_*m*_1__, except that at each time input, instead of giving an element of artic as an output, it gives as an output the set containing that element. Now we can build the silhouette as a representation with sets, i.e., regions, as outputs.

Consider the *n*th iteration of a silhouette; that is, consider ${\text{SIL}}_{c,n}:\left[0,s_{n}\right]\to P\left(\text{ARTIC}\right)$. Suppose that the (*n* + 1)th schema associated with the same concept is schema_*m*_*n*+1__:[0, *s*_*n*+1_] → artic. Let *k* take the value as in criterion 4 above (note that *k* serves an analogous purpose here). Then we find α, β, with 0 ≤ α ≤ β − *k* ≤ *s*_*n*+1_ − *k* such that

$$\begin{array}{l} \frac{1}{\beta -\alpha }\int_{\alpha }^{\beta }\underset{x\in {\text{SIL}}_{c,n}\left(h_{\alpha ,\beta ,s_{n}}\left(t\right)\right)}{\min}\left(d_{\text{ARTIC}}\left(m_{n\;+\;1}\left(t\right),x\right)\right)dt \end{array}$$

equivalently,

$$\begin{array}{l} \frac{1}{s_{n+1}}\int_{0}^{s_{n+1}}\underset{x\in {\text{SIL}}_{c,n}\left(t\right)}{\min}\left(d_{\text{ARTIC}}\left(m_{n\;+\;1}\left(\frac{\beta -\alpha }{s_{n+1}}t+\alpha \right),x\right)\right)dt \end{array}$$

is minimal, where $h_{\alpha ,\beta ,s_{n\left(t\right)}}=\frac{s_{n}}{\beta -\alpha }t-\frac{s_{n}\alpha }{\beta -\alpha }$, analogously to $h_{\alpha ,\beta ,s^{\prime }}$ in criterion 4; that is, we find an alignment of the schema and the silhouette so that the average distance from the schema to the closest point at each time in the silhouette is minimal. More specifically, for each pair of values α and β, this expression aligns the *entire* silhouette with a portion of the schema that runs temporally from α to β and computes the average distance between the two on that stretch. The smaller the average distance, the more appropriate (in some sense) it is to align the silhouette with that piece of the schema. The values of α and β that make this average distance (i.e., the expression above) minimal represent in some sense the optimal alignment of the schema and the silhouette. The success of this procedure (i.e., the minimal value of the expression being satisfactorily small) also entails that the schema necessarily has a portion of it that aligns well with the entire silhouette. This entailment rests on the assumption that words are progressively lengthened by adding on syllables or demisyllables over developmental time (e.g., the production [ˈnænɑ] for “banana” does not follow the production [bəˈnænɑ] in developmental time). Note the similarity of this expression to the expression in criterion 4. In criterion 4, the alignment is essentially required to be good enough (the average distance is required to be “small enough”); whereas, here, the alignment is required to be optimal (that is, the average distance is required to be minimal). Fulfillment of the good enough requirement is sufficient for a motor trajectory to be selected; but when this alignment is being used to build out the silhouette, as described below, it is required to be optimal.

Once the best alignment of a new schema with an existing silhouette is identified, the (*n* + 1)th silhouette for *c*, ${\text{SIL}}_{c,n+1}:\left[0,s_{n+1}\right]\to P\left(\text{ARTIC}\right)$, can be defined:

$$\begin{array}{l} {\text{SIL}}_{c,n+1}(t)\\ =\{\begin{array}{cc} \text{Conv}\left(\left\{{\text{SCHEMA}}_{m_{n+1}}(t)\cup {\text{SIL}}_{c,n}(h_{\alpha ,\beta ,s_{n}}(t))\right\}\right)\cap \text{ARTIC} & \left(\alpha \leq t\leq \beta \right)\\ \left\{{\text{SCHEMA}}_{m_{n+1}}(t)\right\} & \text{otherwise}, \end{array} \end{array}$$

where Conv(*A*) is the convex hull of *A*, for any subset *A* of motor space. (In this case, we consider artic in particular as a subset of an affine space, so the convex hull is defined; see Appendix B).

#### 2.5.3. Adult-Like Production

In Core, adult-like production uses the same process as the second attempt at a word, but a silhouette, rather than a schema, biases the matching and selection process. More precisely, once a silhouette ${\text{SIL}}_{c}:\left[0,s^{\prime }\right]\to P\left(\text{ARTIC}\right)$ exists for a particular concept *c*, a motor trajectory *m*:[0, *s*] → artic and corresponding perceptual trajectory *p*:[0, *s*] → sounds are chosen to communicate *c* based on the three criteria in section 2.5.1, as well as the following criterion, which is a generalization of the criterion 4, the criterion used in the second attempt at a word:

4*. A portion of *m* is close to fitting into the current silhouette for *c*. That is, there exist α and β with 0 ≤ α ≤ β−*k* ≤ *s*−*k* (*k* the same as in the previous criterion 4) such that
  $$\frac{1}{\beta -\alpha }\int_{\alpha }^{\beta }\underset{x\in {\text{SIL}}_{c}\left(h_{\alpha ,\beta ,s^{\prime }}\left(t\right)\right)}{\min}\left(d_{\text{ARTIC}}\left(m\left(t\right),x\right)\right)dt$$

is sufficiently small, where $h_{\alpha ,\beta ,s^{\prime }}$ is as defined in criterion 4.

Importantly, the regions that define the silhouette at each moment along its length will stay the same size with each iteration of a word or increase to include more points. The salience function introduces extensive variability in word production during early child language, which means that the region defined by a silhouette at each point in time will often expand. In addition, the well-grounded assumption that immature motor control introduces noise into execution entails an exploration of motor space adjacent to the planned (selected) motor trajectory. The new paths carved out by this exploration can be purposefully used in future productions to find closer approximations to the perceptual goal. Due to the increasing availability of better approximations, articulatory accuracy increases with developmental time, albeit not necessarily in a linear fashion. Further, we assume that failures in communication are also beneficial to the development of articulatory accuracy in that such failures also define new trajectories through motor space within and adjacent to the regions defined by the silhouettes.

In sum, silhouettes come to represent passages through motor space that are especially well-explored over developmental time. The exploration reticulates the motor space within these passages so completely that the motor phonological representation provides less and less of a constraint on the matching and selection process. Instead, the perceptual constraint can be fully optimized during each production; that is, the perceptual trajectory that is the goal can be closely approximated at each point in time using the set of endogenous perceptual trajectories that are linked to corresponding trajectories in motor space. This is adult-like speech production: a process that is perceptually guided within a silhouette-bounded motoric range.

## 3. Discussion

Intelligible adult speakers achieve language-specific articulatory configurations one after another in rapid sequence. The configurations are typically conceived of as movement in service of speech motor goals. Most adult-focused models of speech production assume that these goals are perceptual or auditory in nature and linked in some manner to a limited set of discrete phonological representations, for example, to phonemes or distinctive features (e.g., Houde and Nagarajan, 2011; Tourville and Guenther, 2011; Hickok, 2012). This assumption introduces a serial order problem that psycholinguistic models of speech production are designed to solve. For at least half a century, the solution has been to posit an encoding process where segmental phonological rules are applied and then phonetic detail is specified (e.g., MacKay, 1970; Dell, 1986; Levelt, 1989; et seq.). Redford (2015, 2019) has argued that this solution is incompatible with a developmental perspective on spoken language production. In particular, the encoding process suggests an acquisition problem too complex to surmount by the time infants are producing first words at 12 months of age. Moreover, the hypothesis is at odds with the sound patterns of early child language, which suggest the whole word as both plan and goal (see, e.g., Vihman and Keren-Portnoy, 2013; Redford, 2019).

A developmental perspective leads us to embrace the alternative to a phonological-phonetic encoding hypothesis; namely, that word forms are remembered and retrieved holistically for production. This whole word production hypothesis solves the serial order problem by avoiding it, but it also begs the question: how does adult-like speech motor control develop absent discrete phonological representations? The Core model provides an answer. The ability to target linguistically significant articulatory configurations one after another in rapid sequence relies on a perceptually guided production process within a silhouette-bounded motoric range subsequent to the emergence of perceptual-motor units, which occurs over developmental time as the motor space becomes increasingly reticulated with exploration.

The central hypothesis in Core that the (near) overlap of motor trajectories yields perceptual-motor units and articulatory chunks for combination implies a production system that is *superficially combinatorial*; that is, a system where “parts of signals overlap (that is, occupy the same position in acoustic and perceptual space) with parts of other signals…Importantly, the overlapping parts of different signals need not necessarily also be the units of combination of the underlying linguistic representation (Zuidema and de Boer, 2009, p. 126).” Zuidema and de Boer distinguish such a system from one that is *productively combinatorial*; that is, a system “where the cognitive mechanisms for producing, recognizing and remembering signals make use of a limited set of units that are combined in many different ways. Productive combinatoriality is a property of the internal representations of language in the speaker (p. 126).” They argue that emergent elements in a superficial combinatorial phonology can become available for use in a productive combinatorial phonology over evolutionary time with communicative pressures. Core demonstrates, however, that the transition from a superficial combinatorial phonological system to a productive one is not necessary to account for normal speech production. Rather, Core assumes phonological representations that are sets of form-meaning pairings. In one set, the forms are holistic, perceptual, and exogenously derived; in the other set, the forms are holistic, motoric, and endogenously derived. Both types of representations are “integrated” for output using the perceptual-motor map according to a matching and selection process that produces increasingly optimal results (i.e., closer matches to the holistic perceptual goal) as the perceptual and motor spaces become increasingly retriculated with vocal-motor exploration and practice. In Core, the matching and selection process may result in a novel motor trajectory that can be analyzed as a combination of smaller paths from multiple trajectories, but there is no sense in which the junctures that delimit these paths are independently recognized and remembered by the speaker to generate a targeted linguistic form.

Although our assertion is that normal speech production is governed by holistic representations, this is not to say that the emergent perceptual-motor units and articulatory chunks posited in Core could not be inducted into the speaker's linguistic system. In fact, we expect that speakers may identify perceptual-motor units and the articulatory chunks they delimit as structurally important linguistic elements with the development of metalinguistic awareness and the right incentives (e.g., the motivation to read and write). This identification may never be critical to the speech production process, but could be useful for creative language, including for rhyming and for creating lines that are onomatopoetic, alliterative, and so on. We suggest that both the identification of perceptual-motor units as elements of linguistic structure and the creative use of these elements in spoken or written verse rely on a speaker's intuition of sound/action equivalence, which is in turn grounded in notions of perceptual and articulatory distance. These notions are themselves based on metrics implied in the architecture of motor and perceptual spaces in Core.

Specifically, one can define a distance metric on the set of equivalence classes of motor trajectories that aligns with the structures described in Core. Let *m*:[0, *s*] → artic and *m*′:[0, *s*′] → artic be motor trajectories. Define the distance between them to be

(i) $$\begin{array}{r} \int_{0}^{1}d_{\text{ARTIC}}\left(m\left(st\right),m^{\prime }\left(s^{\prime }t\right)\right)dt \end{array}$$

It can be checked that this is a pseudometric on the set of motor trajectories; that is, it is nearly a metric, except for the fact that there are (in theory) trajectories that are a distance of zero from each other that are nevertheless distinct due to global timing differences. The equivalence relation defined in section 2.2.3 treats two such trajectories as equivalent. The pseudometric then induces a metric on the set of equivalence classes; that is, the metric is compatible with the structure on the set of motor trajectories that has been laid out. For example, one can easily observe the similarity between this metric and the way that the distance between a motor trajectory and a motor silhouette is measured. Consider a case where the expression in criterion 4* is utilized to compare a motor trajectory *m*:[0, *s*] → artic and a silhouette ${\text{SIL}}_{c}:\left[0,s^{\prime }\right]\to P\left(\text{ARTIC}\right)$, specifically with the alignment that compares the entirety of the motor trajectory to the entirety of the silhouette. That expression in this case becomes

$$\frac{1}{s}\int_{0}^{s}\underset{x\in {\text{SIL}}_{c}\left(s^{\prime }t/s\right)}{\min}d_{\text{ARTIC}}\left(m\left(t\right),x\right)dt,$$

which is equal to

$$\int_{0}^{1}\underset{x\in {\text{SIL}}_{c}\left(s^{\prime }t\right)}{\min}d_{\text{ARTIC}}\left(m\left(st\right),x\right)dt$$

through a change of variables. Then, let *m*′:[0, *s*′] → artic be a theoretical motor trajectory that is the closest possible at each point in time to *m*, while still being contained in the motor silhouette sil_*c*_ (i.e. *m*′(*t*) is in sil_*c*_(*t*) for each *t*). This expression is then equivalent to

$$\int_{0}^{1}d_{\text{ARTIC}}\left(m\left(st\right),m^{\prime }\left(s^{\prime }t\right)\right)dt,$$

which is the distance between motor trajectories *m* and *m*′ as just defined in (i). In other words, using the procedure described in criterion 4* to compare a motor trajectory to a silhouette on the entirety of both of their domains is equivalent to comparing that motor trajectory to a theoretical closest motor trajectory that is contained in the silhouette. It is in this way that these two notions are compatible. The relationship of (i) to the expression in criterion 4 (being a special case of the expression in criterion 4*) is even more straightforward—if α and β are set to be 0 and *s*_1_, respectively, then the expression in criterion 4 is exactly the expression (i) applied to motor trajectories *m*_1_ and *m*′.

Similarly, let *p*:[0, *T*] and *p*′:[0, *T*′] be two perceptual trajectories (self-productions and/or exemplars). It is reasonable to define the distance between them to be the sum, or in this case average (which can be seen as a time-normalized sum), of the distances between them at each time (Itakura, 1975, p. 69; Mermelstein, 1976; Sakoe and Seibi, 1978; inter alia). More specifically, define the distance between them to be

(ii) $$\begin{array}{r} \int_{0}^{1}d_{\text{SOUNDS}}\left(p\left(Tt\right),p^{\prime }\left(T^{\prime }t\right)\right)dt. \end{array}$$

As in the motor case, this is a pseudometric on the set of perceputal trajectories that yields a metric on the set of equivalence classes of perceptual trajectories defined in section 2.2.3. Moreover, observe that this is the same as the measure between a self-production and a perceptual trajectory as defined in criterion 2 if the salience were 1 everywhere—that is, if the whole of the exemplar were fully salient—as would likely be the case in adult speech. Thus, this metric is a good representation of the structure on the set of perceptual trajectories for an adult (for a discussion of desirable properties of perceptual distance measures, see Mermelstein, 1976).

A psychological notion of distance could emerge from the implied metrics described above. This notion could then account for the experience of two words as sounding or feeling similar. A creative language behavior, like rapping, could then be understood as the conscious exploitation of an intrinsic matching algorithm; specifically, as an attempt at minimizing the perceptual trajectory distance between two word-length perceptual trajectories, and/or minimizing the motor trajectory distance between two word-length motor trajectories; or as an attempt at keeping these distances within a certain range. For example, the impression that a line flows well in a rap might because the speaker has identified perceptual trajectories that are similar enough that the distance between them is below a certain threshold, but are different enough that they are not pure repetition (e.g. Eminem's “…all the stores ship us platinum” and then “…metamorphosis happen”; Mathers et al., 2002, track 12). Rhyming, on the other hand, is a particular instantiation of bounding the distance between perceptual trajectories, wherein a not-too-large, not-too-small average distance between trajectories is achieved specifically by making the distances larger at the onset, and very small in the rhyme. This additional restriction would require modulation or deliberate new constraints on the perceptual matching algorithm that is intrinsic to Core.

The distance metrics we define are fundamental to speech production and development in Core because both rely on comparisons between trajectories. Two critical comparison operations are matching to approximate a phonetically detailed perceptual representation (i.e., an exemplar) to produce words, and matching existing schemas to create an abstract motor phonological representation (i.e., a silhouette). The algorithms we instantiate to effect these and other comparison operations were sometimes motivated by specific hypotheses regarding spoken language behavior; other times they were expedient. For example, a theoretically motivated assumption underlies the choice to represent perceptual trajectories that are exemplars and perceptual trajectories that are self-generated in the same perceptual space and then match them based on patterns (e.g., the difference between *Z*_1_ and *Z*_3_) rather than based on absolute values (e.g., the values of *Z*_1_ and *Z*_3_). The assumption is that infants do not track the various acoustic correlates to linguistic contrasts separately; rather, they attend to how the correlates covary in time (see, e.g., Sussman, 1986). This assumption implies that the normalization problem is not actively solved during development. Instead, it is automatically solved in speech processing and production (for a contrasting view see, e.g., Plummer, 2014).

In contrast to the representation of perceptual trajectories, the choice to consider two trajectories equivalent if one can be made into the other by uniform stretching was merely expedient. A more accurate model would include a more nuanced method for the direct comparison of two perceptual or motor trajectories. In particular, applying non-linear time warping might be preferable to the uniform stretching algorithm we used here, since it would more readily capture the disproportionate changes that vowels undergo relative to consonants with changes in speech rate (e.g., Gay, 1981). Techniques used in functional data analysis (see, e.g., Ramsay and and Silverman, 2002) or dynamic time warping algorithms (see, e.g., Sakoe and Seibi, 1978; Furui, 1986) could be considered for this^21^; however, many, if not all, dynamic time warping algorithms do not yield perfect metrics (Casacuberta et al., 1987), which is a disadvantage for defining distance in the perceptual and motor spaces. On the other hand, it may be the case that there exist dynamic time warping methods whose outcomes are essentially metrics on the set of *actual* vocalizations, which is a subset of the set of theoretically possible vocalizations (ibid).

There are a number of other examples of expedient choices that we made when formalizing the model. The most notable of these are the many criteria that were left underspecified. For example, in criterion 2 and criteria 4 and 4^*^, a particular measure of distance is required to be “small enough” or “sufficiently small.” We also choose trajectories that “best fulfill” criteria 2, 3, and 4, but we do not specify what optimal fulfillment means. These underspecified criteria suggest avenues for future research. For example, when a quantity is “small enough,” that could mean it lies below some threshold value that is either fixed or changing over developmental time. Alternatively, “small enough” could mean “smallest out of some comprehensive set of objects considered”. These and other open questions could be answered in empirical research designed to test different model-based predictions.

## Author Contributions

The paper is fully collaborative. Each author contributed 50% effort to the manuscript. MD's primary responsibility was to formalize the model. MR's primary responsibility was to conceptualize the model. Both authors contributed to the writing.

### Conflict of Interest Statement

The authors declare that the research was conducted in the absence of any commercial or financial relationships that could be construed as a potential conflict of interest.

## Footnotes

^1^This statement is consistent with the modern conception of the serial order problem, but it mischaracterizes Lashley's (1951) argument. Lashley proposed that complex skilled action is effected with reference to a control structure of hierarchically-arranged discrete action units. In other words, the problem that Lashley addressed was that of complex skilled action; his solution introduced the serial order problem as it is currently conceived.

^2^Gestures are abstract representations of linguistically-significant vocal tract constrictions, similar to distinctive features but with intrinsic timing; for example, a labial vs. alveolar gesture gives rise to the minimal pair “bog” vs. “dog” where these gestures are co-produced (temporally overlapped) with the gesture associated with the following vowel.

^3^The processes modeled in Core are nonetheless compatible in principle with a dynamical systems approach to this separate question of articulatory coordination (e.g., Saltzman and Munhall, 1989; Sorensen and Gafos, 2016).

^4^As this example suggests, the distinction we make between linguistic and non-linguistic depends on the functional definition of language as a system of form-meaning pairs (see also Saussure, 1959; Langacker, 1987; Fillmore et al., 1988; Goldberg, 1995; Bybee, 2001; Croft and Cruse, 2004; inter alia).

^5^It could equally be the case that an infant initially remembers only the most salient portions of a word (see, e.g., Vihman, 2017), and that the exemplar representation therefore changes with developmental time.

^6^See section 2.2.3 for details.

^7^See Snow (1998) for a related prominence account of weak syllable deletion.

^8^The idea that familiar items become less salient with repeated attention is based on the well-studied relationship between habituation and the emergence of a novelty preference in infant studies (see, e.g., Sirois and Mareschal, 2002).

^9^The emphasis on communicative success for schema formation is consistent with the recent revival of interest in associative learning for understanding speech and language acquisition (e.g., Howard and Messum, 2011; Ramscar et al., 2013; Warlaumont and Finnegan, 2016; see also Kapatsinski, 2018).

^10^Although the perceptual-motor map may or may not be a true bijection, the insights we offer from this model are not dependent on this particular assumption. Instead they depend on the assumption that motor and perceptual trajectories are systematically linked to one another.

^11^Clearly sound requires time and so “instantaneous sound” should not be interpreted as psychologically real. Instead, the construct is simply used to formalize the idea of trajectories. In fact, we never treat sound as independent of the trajectory on which it lies. In this way, all sound (and for that matter, movement) is inseparable from time in the Core model.

^12^We use phon values and Bark-transformed values instead of the more familiar RMS pressure and Hertz values to code loudness and formant frequency information in order to underscore the point that the dimensions we seek to define are psychological, not physical. The reader should imagine that the spectral center of gravity and the width of the spectral peak are similarly transformed from the physical to the psychological.

^13^We assume that these variables are modeled well as piecewise continuous functions of time that are differentiable almost everywhere (i.e., on all but a set of measure zero). If at a particular point in time the derivative of one of these variables does not exist, we set it to zero to give it a well-defined value.

^14^This choice clearly elides the problem of articulatory movement and coordination that is central to other models of speech motor control (e.g., Saltzman and Munhall, 1989; Guenther, 1995, 2016), but is in keeping with our specific interest in the relationship between phonological representation and motor control. The choice is nonetheless plausibly motor in that the cross-sectional area of the vocal tract can presumably be recovered from somatosensory feedback. It is in this way that the motor space in Core resembles the somatosensory space/reference frame in DIVA.

^15^The Bark Difference Metric is a vowel-intrinsic normalization method adapted from Syrdal and Gopal (1986). Perceptual distance is normalized for speaker differences based on our assumption that exogenously-derived exemplars are trajectories in the same perceptual space as the trajectories that are auditory memories of self-productions, which define the perceptual aspect of the perceptual-motor map.

^16^It can be checked that this is in fact an equivalence relation.

^17^To be even more specific in reference to the Appendix, in this example we have ℓ = 2 because we are only using two “paths” to build our new trajectory; *k*_1_ = *m*_1_ and *k*_2_ = *m*_2_ are the trajectories *m* is being built out of; α_1_ = 0, β_1_ = 0.6, α_2_ = 0.7, and β_2_ = 2, meaning the intervals on which we are using *k*_1_ and *k*_2_ (in this case, *m*_1_ and *m*_2_) are [0, 0.6] and [0.7, 2].

^18^Recall that salience is defined by an infant's attention, which, though not determined by acoustic properties, is nonetheless influenced by them such that, for example, louder and longer sounds are expected to be more salient than quieter and shorter sounds. Of course, defined in this way, salience is likely to affect the acquisition and representation of exemplars. This means that perceptual phonological representations are as likely to change through developmental time as motor phonological representations. In Core, we gloss over this implication for the sake of simplicity. In lieu of modeling developmental changes to exemplar representations, we only model salience at the moment of production. Put another way, a more complete model would include the effect of salience on initial exemplar representation (i.e., a salience “filter” on the perceptual input) in addition to salience as internally directed attention to the perceptual goal.

^19^See Appendix B for the definition of a convex hull.

^20^Taking the power set is a necessary technical detail; see Appendix B for the rigorous definition of the convex hull, which requires a set as an input.

^21^See Mermelstein (1976) for a review of different distance measures.
