# Supplementary material for: The Emergence of Discrete Perceptual-Motor Units in a Production Model That Assumes Holistic Phonological Representations
Source: Front Psychol. 2019 Sep 18;10:2121. doi: 10.3389/fpsyg.2019.02121 (PMC6759877; doi:10.3389/fpsyg.2019.02121)
Supplement: Supplementary file 1 [file Data_Sheet_1.pdf]

1 Appendix A

Let $\{m_1, \ldots, m_n\}$ be the set of motor trajectories that have been executed (and therefore stored in the motor trajectory set). Suppose they have $s_1, \ldots, s_n$ open-close cycles, respectively. Thus, their domains are $[0, s_1], \ldots, [0, s_n]$, respectively. The following rule describes what new motor trajectories can be made out of the existing ones. First, we define a parameter ε to take some value greater than 0. This will be the maximum distance within the articulatory configuration space that the speaker can shift to get from one trajectory to another.

A motor trajectory $m : [0, s] \to \text{ARTIC}$ (that is, with s open-close cycles) can be created from the existing motor trajectory set if there exist $k_1, \ldots, k_\ell \in \{m_1, \ldots, m_n\}$ (not necessarily distinct), and subintervals of their respective domains, $[\alpha_i, \beta_i] \subset [0, s_i]$ for each i such that m can be defined in terms of $k_1, \ldots, k_\ell$ in the following way:

$$m(t) = \begin{cases} k_1\left(\alpha_1 + \frac{t}{u}\right) & \text{if } 0 \le t \le u(\beta_1 - \alpha_1) \\ (1 - \lambda_1(t))k_1(\beta_1) + \lambda_1(t)k_2(\alpha_2) & \text{if } u(\beta_1 - \alpha_1) \le t \le u(\beta_1 - \alpha_1 + \delta_1) \\ k_2\left(\alpha_2 + \frac{t}{u} - (\beta_1 - \alpha_1 + \delta_1)\right) & \text{if } u(\beta_1 - \alpha_1 + \delta_1) \le t \\ & \qquad \le +u(\beta_1 - \alpha_1 + \delta_1) + u(\beta_2 - \alpha_2) \\ (1 - \lambda_2(t))k_2(\beta_2) + \lambda_2(t)k_3(\alpha_3) & \text{if } u\delta_1 + u\sum_{i=1}^{2}(\beta_i - \alpha_i) \le t \\ & \qquad \le u\sum_{i=1}^{2}\delta_i + u\sum_{i=1}^{2}(\beta_i - \alpha_i) \\ \vdots & \vdots \\ k_j\left(\alpha_j + \frac{t}{u} - \sum_{i=1}^{j-1}(\beta_i - \alpha_i + \delta_i)\right) & \text{if } u\sum_{i=1}^{j-1}(\beta_i - \alpha_i + \delta_i) \le t \\ & \qquad \le u\sum_{i=1}^{j-1}(\beta_i - \alpha_i + \delta_i) + u(\beta_j - \alpha_j) \\ (1 - \lambda_j(t))k_j(\beta_j) + \lambda_j(t)k_{j+1}(\alpha_{j+1}) & \text{if } u\sum_{i=1}^{j-1}\delta_i + u\sum_{i=1}^{j}(\beta_i - \alpha_i) \le t \\ & \qquad \le u\sum_{i=1}^{j}\delta_i + u\sum_{i=1}^{j}(\beta_i - \alpha_i) \\ \vdots & \vdots \\ (1 - \lambda_{\ell-1}(t))k_{\ell-1}(\beta_{\ell-1}) + \lambda_{\ell-1}(t)k_\ell(\alpha_\ell) & \text{if } u\sum_{i=1}^{\ell-2}\delta_i + u\sum_{i=1}^{\ell-1}(\beta_i - \alpha_i) \le t \\ & \qquad \le u\sum_{i=1}^{\ell-1}\delta_i + u\sum_{i=1}^{\ell-1}(\beta_i - \alpha_i) \\ k_\ell\left(\alpha_\ell + \frac{t}{u} - \sum_{i=1}^{\ell-1}(\beta_i - \alpha_i + \delta_i)\right) & \text{if } u\sum_{i=1}^{\ell-1}(\beta_i - \alpha_i + \delta_i) \le t \\ & \qquad \le u\sum_{i=1}^{\ell-1}(\beta_i - \alpha_i + \delta_i) + u(\beta_\ell - \alpha_\ell) \ \ (= s), \end{cases}$$

where u, λ_j, and δ_i are defined in the following way, and the criteria (*), (**), and (***) below are met. We set $u = \dfrac{s}{\sum_{i=1}^{\ell}(\beta_i - \alpha_i) + \sum_{i=1}^{\ell-1}\delta_i}$, which acts as a stretching factor to ensure that m has the appropriate domain. Ideally, u would be close to one, since the number of open-close cycles of m should be close to the sum of the numbers of open-close cycles in each of the pieces trajectories used to build m, i.e. the denominator in the expression for u should be close to s. To define δ_i, a global parameter, v, is defined. This is our rate parameter – something like the normalized velocity

with which the speaker moves through open-closed cycles. Then for each i, we define

$$\delta_i = \frac{d_{\text{ARTIC}}\left(k_i\left(\beta_i\right), k_{i+1}\left(\alpha_{i+1}\right)\right)}{v},$$

where d is the distance function on the articulatory configuration space as a metric space. This means δ_i can be thought of as the normalized amount of time it will take, assuming a particular normalized velocity, to make the shift from the ith path to $(i+1)$th path. Finally, define

$$\lambda_j(t) = \frac{1}{u\delta_j}t - \frac{\sum_{i=1}^{j}(\beta_i - \alpha_i) + \sum_{i=1}^{j-1}\delta_i}{\delta_j}.$$

This is a variable that changes linearly with time that simply ensures that m's path goes in a "straight line" at rate v when shifting from path to path. It seems likely that this is not an accurate representation of movement, but it is a simplifying assumption that only affects small portions of the path.

We also require that the numerator in the equation defining δ_i satisfy the following criterion for all i:

$$d_{\text{ARTIC}}\left(k_i\left(\beta_i\right), k_{i+1}\left(\alpha_{i+1}\right)\right) < \varepsilon. \qquad (\star)$$

That is, the distance the speaker can shift between each pair of trajectories must be smaller than ε. Additionally, we require that

$$(^{\star\star}) \qquad \alpha_1 = 0 \qquad \text{and} \qquad (^{\star\star\star}) \qquad \beta_\ell = s_\ell.$$

That is, the piece of the first trajectory that is used must include the beginning of the trajectory, and the piece of the last trajectory must include the end of that trajectory.

2 Appendix B

To define the notion of a *convex hull*, we start with an *affine space*, which we will not define here, but can be imagined with reference to familiar examples of affine spaces, such as Euclidean space or any vector space – although these both have additional structure on top of being affine spaces. The important part about an affine space in the context of a convex hull is that if one has a pair of points, there is a notion of which other points in the space lie *between* those the points; that is, there is a notion of a line segment between the two points. The reason we need the notion of "between" is because the (not quite rigorous) definition of a convex set is the following: if X is an affine space, and Y is a subset of X, then Y is convex if for every pair of points in Y, every point between those two points in the space X are also in Y. Recalling the familiar notion of convex polygons (which exist in 2-dimensional Euclidean space), one can realize that indeed, for any two

points in a convex polygon (including on the boundary), every point between them is also in the polygon (perhaps on the boundary).

Let A be a subset of our affine space. Then the *convex hull of A*, denoted $\mathrm{Conv}(A)$, is the minimal convex subset of the affine space that contains every element of A. This notion is best described through illustration. Suppose that we consider the space on this page to belong to 2-dimensional Euclidean space. Then we can consider the convex hulls of various subsets (collections of points) of this space. Illustrations of this are shown in Figure 1.

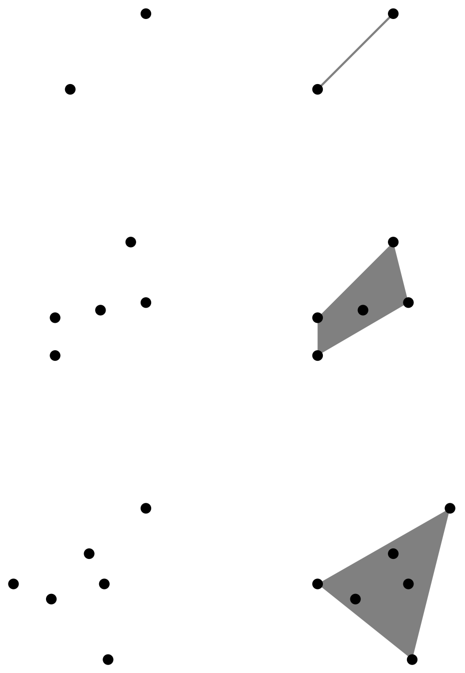

Figure 1: Three subsets of 2-dimensional Euclidean space, and their respective convex hulls, are shown, with each subset on the left, and its convex hull on the right.

We consider motor space to be a subset of an affine space, where the affine space is the set of 11-tuples of real numbers with an affine structure defined in the normal way (coordinate-wise subtraction, etc.). The reason we choose to assume motor space is a subset of an affine space rather than making the stronger assumption that it is a subset of a vector space is that the meaning of the elements of motor space are more aligned with the framework of an affine space. For example, one does not really want to think about "adding" two articulatory configurations to each other, an operation which is embedded into the definition of a vector space. However, we do want to be able to represent the *difference* between two articulatory configurations with a vector, and we want to be able to add and scale those difference vectors.

Let E be the affine space in which motor space is embedded. Let A be a subset of motor space. Then we define $\mathrm{Conv}(A)$ to be the convex hull of A *in E*. This means that $\mathrm{Conv}(A)$ might contain

some elements that are not motorically possible. To alleviate this issue, whenever we take the convex hull of a subset of motor space, we then take its intersection with the set of points that constitute motor space, i.e. $\text{Conv}(A) \cap \textsc{artic}$. This is the motorically possible subset of the convex hull of A.

3 Appendix C

$f : A \to B$	Read "f from A to B"; implicitly states that f is a function with domain A and codomain B (so A and B are implicitly sets)
ARTIC	The set of all possible articulatory configurations, along with the metric and topology described
PERC	The set of all possible instantaneous sounds, along with the metric and topology assumed to exist
d_{ARTIC}	The distance metric on the set of articulatory configurations (specified in the text)
d_{SOUNDS}	The distance metric on the set of instantaneous sounds (not specified)
$d_{\text{ARTIC}}(a_1, a_2)$	The distance between articulatory configurations a_1 and a_2, according to the metric d_{ARTIC}
$d_{\text{SOUNDS}}(s_1, s_2)$	The distance between instantaneous sounds s_1 and s_2, according to the metric d_{SOUNDS}
$\min_{x \in A}(f(x))$	The smallest value that $f(x)$ takes where x is an element of A. For example, $\min_{x \in \{-3,2,5,10\}}(x^2) = 4$.
Convex hull, denoted Conv	See Appendix B
$A \cap B$	The *intersection* of the sets A and B; that is, the set of elements that are in both A and B. For example, if $A = \{1, 2, 8\}$ and $B = \{2, 8, -3\}$, then $A \cap B = \{2, 8\}$.
$A \cup B$	The *union* of the sets A and B; that is, the set of elements that are in A or B (including elements that are in both). For example, if $A = \{1, 2, 8\}$ and $B = \{2, 8, -3\}$, then $A \cup B = \{1, 2, 8, -3\}$.
